# The Complete Genome Sequence of the Fish Pathogen *Tenacibaculum maritimum* Provides Insights into Virulence Mechanisms

**DOI:** 10.3389/fmicb.2017.01542

**Published:** 2017-08-16

**Authors:** David Pérez-Pascual, Aurelie Lunazzi, Ghislaine Magdelenat, Zoe Rouy, Alain Roulet, Celine Lopez-Roques, Robert Larocque, Tristan Barbeyron, Angélique Gobet, Gurvan Michel, Jean-François Bernardet, Eric Duchaud

**Affiliations:** ^1^Virologie et Immunologie Moléculaires, Institut National de la Recherche Agronomique, Université Paris-Saclay Jouy-en-Josas, France; ^2^Commissariat à l’Énergie Atomique et aux Énergies Alternatives, Institut de Génomique, Genoscope Evry, France; ^3^Laboratoire d’Analyses Bioinformatiques en Génomique et Métabolisme, Centre National de la Recherche Scientifique (UMR-8030), Commissariat à l’Énergie Atomique et aux Énergies Alternatives, Institut de Génomique, Genoscope Evry, France; ^4^Genotoul Genome & Transcriptome (GeT-PlaGe), Institut National de la Recherche Agronomique Castanet-Tolosan, France; ^5^Institut National de la Recherche Agronomique (UAR1209) Castanet-Tolosan, France; ^6^Laboratoire de Biologie Intégrative des Modèles Marins (UMR 8227), Centre National de la Recherche Scientifique, Université Pierre et Marie Curie, Station Biologique de Roscoff, Sorbonne Universités Roscoff, France

**Keywords:** *Tenacibaculum maritimum*, fish pathogen, virulence factors, genome, toxins

## Abstract

*Tenacibaculum maritimum* is a devastating bacterial pathogen of wild and farmed marine fish with a broad host range and a worldwide distribution. We report here the complete genome sequence of the *T. maritimum* type strain NCIMB 2154^T^. The genome consists of a 3,435,971-base pair circular chromosome with 2,866 predicted protein-coding genes. Genes encoding the biosynthesis of exopolysaccharides, the type IX secretion system, iron uptake systems, adhesins, hemolysins, proteases, and glycoside hydrolases were identified. They are likely involved in the virulence process including immune escape, invasion, colonization, destruction of host tissues, and nutrient scavenging. Among the predicted virulence factors, type IX secretion-mediated and cell-surface exposed proteins were identified including an atypical sialidase, a sphingomyelinase and a chondroitin AC lyase which activities were demonstrated *in vitro*.

## Introduction

*Tenacibaculum maritimum* (formerly *Flexibacter maritimus*), a member of the family Flavobacteriaceae, phylum Bacteroidetes (Suzuki et al., 2001), is the etiological agent of tenacibaculosis, a very serious bacterial disease of many commercial marine fish species (for a review, see Avendaño-Herrera et al., 2006b), responsible for considerable economic losses in all major areas of marine finfish aquaculture worldwide (i.e., Japan, Europe including the Atlantic, Channel and Mediterranean coasts, North America, Australia, and the Red Sea). Moreover, *T. maritimum* can affect a large number of feral, captive, and cultured fish species such as: Dover sole (*Solea solea*), Senegalese sole (*Solea senegalensis*), wedge sole (*Dicologoglossa cuneata*), turbot (*Scophthalmus maximus*), Atlantic salmon (*Salmo salar*), Japanese flounder (*Paralichthys olivaceus*), yellowtail (*Seriola quinqueradiata*), red sea bream (*Pagrus major*), black sea bream (*Acanthopagrus schlegelii*), gilthead sea bream (*Sparus aurata*), European sea bass (*Dicentrarchus labrax*), puffer fish (*Takifugu rubripes*), Pacific sardine (*Sardinops sagax*), lumpsucker (*Cyclopterus lumpus*), and sand tiger shark (*Carcharias taurus*) (Bernardet et al., 1990; Avendaño-Herrera et al., 2006a; López et al., 2009; AbdEl-Galil and Hashiem, 2011; Rahman et al., 2014; Florio et al., 2016; Småge et al., 2016; and references therein]. Affected fish usually display a variety of external signs including eroded mouth, skin ulcers, fin necrosis, and tail-rot. Skin lesions are often colonized by opportunistic pathogens such as *Vibrio* spp. So far, only one specific vaccine is commercially available to prevent tenacibaculosis in turbot. Hence, in all other fish species, the control of tenacibaculosis outbreaks remains restricted to the use of antibiotics, sometimes combined with external disinfectants (Avendaño-Herrera et al., 2008).

So far, three serotypes have been documented that show varying degrees of association with host fish species (Avendaño-Herrera et al., 2005a). This serological diversity could have important consequences for the development of an efficient vaccine. Recently, multilocus sequence analysis (MLSA) of *T. maritimum* isolates representative of the worldwide diversity revealed that this species constitutes a cohesive group, exhibiting moderate levels of nucleotide diversity and recombination [average pairwise nucleotide diversity (π) estimated to be 0.44% and *r/m* ratio estimated to be 2.7]. Moreover, the population structure of *T. maritimum* did not reveal dominant genotypes or clonal complexes but rather suggested an endemic colonization of fish farms by local strains with no contribution of long-distance contamination related to fish movements. In addition, the same MLSA genotype was identified in different host species in the same geographical area, suggesting host versatility (Habib et al., 2014).

Despite the significance of tenacibaculosis outbreaks in the aquaculture industry, little is known about the virulence mechanisms of *T. maritimum* (Avendaño-Herrera et al., 2006b). Adhesion to hydrophobic surfaces (Burchard et al., 1990) or fish skin mucus (Magariños et al., 1995), hemagglutination (Pazos, 1997), extracellular products including proteolytic activity (Baxa et al., 1988; Handlinger et al., 1997; Pazos, 1997; van Gelderen et al., 2009), and iron uptake mechanisms (Avendaño-Herrera et al., 2005b) have been suggested to play roles in virulence. However, the molecular factors involved remain to be identified. Loss-of-function studies for experimental validation of genes as virulence factors are still inaccessible due to the absence of genetic tools.

In the present work, we sequenced and analyzed the complete genome of *T. maritimum* NCIMB 2154^T^ to forecast the genes relevant to the bacterial lifestyle, in particular those linked to virulence. These *in silico* predictions paved the way for assessing for the first time the functional role of some relevant components. This genome will serve as a reference for future whole genome-based molecular epidemiology surveys aimed at analyzing disease emergence and propagation (Bayliss et al., 2017).

## Materials and Methods

### Bacterial Growth Conditions

Several batches of the *T. maritimum* type strain (i.e., NCIMB 2154^T^, ATCC 43398^T^, CIP 103528^T^, and DSM 17995^T^), *Tenacibaculum discolor* LL04 11.1.1^T^, *Tenacibaculum jejuense* CNURIC013^T^, and *Tenacibaculum soleae* LL04 12.1.7^T^ were routinely grown in marine broth and agar 2216 (Difco) at 28°C and 170 rpm.

### Genome Sequencing

*Tenacibaculum maritimum* NCIMB 2154^T^ was sequenced with a combination of PacBio RSII (N50 reads 7.4 kb, estimated coverage 234 x) and Illumina (HiSeq 2x100 pair-end reads with 300 bp insert size, 54,259,876 filtered sequences, estimated coverage 1500 x) reads and assembled with MHAP to completion to obtain a circular molecule. The final, quiver polished assembly was validated by optical mapping using *Nco*I.

### Annotation and Genome Comparisons

Genome annotation, including manual curation, and comparisons were performed using the web interface MicroScope (Vallenet et al., 2013) which allows graphic visualization enhanced by a synchronized representation of synteny groups^1^. Predictions of repeated sequences were performed using Repseek (Achaz et al., 2007) and those of genomic islands (GIs) using SIGI-HMM (Waack et al., 2006) and Alien hunter (Vernikos and Parkhill, 2006). The dbCAN database was used to identify carbohydrate active enzymes (CAZymes)^2^ (Yin et al., 2012). The genomic sequence reported in this article has been deposited in the EMBL database under the accession number LT634361.

### MLST on Selected Strains

The four above-mentioned batches of the *T. maritimum* type strain were genotyped using the MLST scheme described in Habib et al. (2014).

### Chondroitin AC Lyase and Sphingomyelinase Cloning, Expression, and Enzymatic Activity

The genes encoding the chondroitin AC lyase (*cslA*, locus identifier: *MARIT_2107*) and sphingomyelinase (*sph*, locus identifier: *MARIT_1748*) were cloned according to Groisillier et al. (2010). Briefly, primers were designed to amplify the coding region corresponding to the catalytic module of CslA (forward primer 5′-TTTTTTAGATCTACTTCTCTAACTTTGGATGTAAATTCG-3′; reverse primer 5′-TTTTTTGAATTCTTATATTTTAAGAACTTTCTCTGTTATTAG-3′) and *sph* (forward primer 5′-AAAAAAGGATCCAATGATGACGTTTCCCTTGGAGAAA-3′; reverse primer 5′-TTTTTTCAATTGTTAGTAGCTAAAGTAAAAAGTTTGCTTG-3′) by PCR from *T. maritimum* genomic DNA. After digestion with the restriction enzymes *Bgl*II and *Eco*RI, and *Bam*HI and *Mfe*I respectively, the purified PCR products were ligated using the T4 DNA ligase into the expression vector pFO4 predigested by *Bam*HI and *Eco*RI (referred to as the plasmid pCslA and psph), resulting in a recombinant protein with a N-terminal hexa-histidine tag for each construct. The obtained plasmids were transformed into *Escherichia coli* DH5α for storage and in *E. coli* BL21(DE3) for protein expression. *E. coli* BL21(DE3) cells harboring the plasmid pCslA or psph were cultivated at 20°C in a 3 mL auto-induction ZYP 5052 medium (Studier, 2005) supplemented with 100 μg/mL ampicillin. Cultures were stopped after 72 h and centrifuged for 35 min at 4°C, 3,000 *g*. The cells were resuspended in 500 μL of buffer A (20 mM sodium phosphate pH 7.4, 500 mM NaCl, 10 mM imidazole). An anti-proteases mixture (cOmplete^TM^ EDTA-free, Roche) and 0.1 mg/mL of DNase were added. The cells were disrupted by sonication. After centrifugation at 12,500 *g* for 2 h at 4°C the supernatant was loaded onto a His spin trap column (GE Healthcare Life Science) equilibrated with buffer A. After extensive washing with buffer A, the recombinant proteins were eluted with 400 μL of buffer B (20 mM sodium phosphate pH 7.4, 500 mM NaCl, 500 mM imidazole). The results were analyzed by 12 % sodium dodecyl sulfate-polyacrylamide gel electrophoresis.

### Chondroitin AC Lyase *In Vitro* Activity

The native chondroitin AC lyase activity of *T. maritimum* was assayed *in vitro* according to Li et al. (2015). Briefly, 6 μL of mid-log-phase bacterial cultures (OD_600_ = 0.6) were spotted on marine agar 2216 supplemented with 0.2% chondroitin sulfate A or C (Sigma) and 2% bovine serum albumin (BSA, Sigma), and incubated for 48 h at 28°C. Chondroitin lyase activity was visualized as a clear halo surrounding the bacterial growth after the plates were flooded with 0.35 N HCl.

The activity of the recombinant CslA was determined by adding 1 μL of the recombinant protein solution to a 1-mL cuvette containing 600 μL of 50 mM Tris–HCl, pH 8.0 supplemented by 1 mg/mL of chondroitin A or C at 30°C. Product formation was monitored as an increase in absorbance at 232 nm as a function of time (Michel et al., 2004). The assay was performed in triplicate.

### Sphingomyelinase Activity

The activity of the recombinant Sph was determined using a coupled assay Amplex Red Sphingomyelinase assay kit (Life Technologies, Invitrogen) following the manufacturer’s instructions. The reactions were performed in 96-well special optics flat clear bottom black polystyrene Microplates (Corning). The reaction mixture (200 μL) contained 100 μL of 1.3 and 13 ng of recombinant protein and 100 μL of 100 μM Amplex red reagent (containing 2 U/mL horseradish peroxidase, 0.2 U/mL choline oxidase, 8 U/mL alkaline phosphatase, and 0.5 mM sphingomyelin). The fluorescence was measured every minute at excitation and emission wavelengths of 530 nm and 590 nm, respectively, using the TECAN Infinite^®^ Pro200 microplate reader at 28°C for 15 min. The background fluorescence was corrected by subtracting the negative control (i.e., without recombinant protein). The positive control of each experiment was performed with *Bacillus cereus* sphingomyelinase provided by the manufacturer. The assay was performed in triplicate.

### Sialidase Activity

The fluorogenic substrate 2′-(4-methylumbelliferyl)-α-D-*N*-acetylneuraminic acid sodium salt hydrate (MUAN) was used to determine sialidase activity according to Mally et al. (2008). Briefly, 10^7^ mid-log phase bacteria were incubated with 0.1 mM of MUAN (Sigma) in 100 mM sodium acetate buffer pH 7.4 at 28°C. The reaction was stopped by the addition of a 0.5 M Na_2_CO_3_ solution at pH 10. Released 4-methylumbelliferone was measured by fluorescence in a TECAN Infinite^®^ Pro200 microplate reader at an excitation wavelength of 360 nm and an emission wavelength of 440 nm. The background fluorescence was corrected by subtracting the negative control (i.e., without bacteria). The assay was performed in triplicate.

## Results and Discussion

### General Genome Features

**FIGURE 1:**
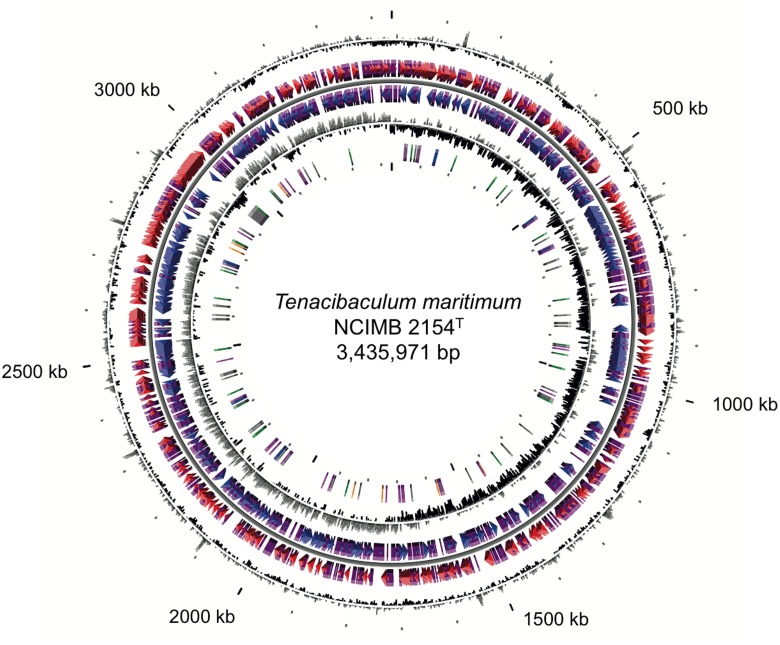
Circular representation of the *Tenacibaculum maritimum* NCIMB 2154^T^ genome. Circles display (from the outside): (1) GC percent deviation (GC window – mean GC) in a 1,000-bp window. (2) Predicted CDSs transcribed in the clockwise direction. (3) Predicted CDSs transcribed in the counterclockwise direction. Genes displayed in (2) and (3) are color-coded according to the following categories: red and blue, MaGe validated annotations; purple, primary/automatic annotations. (4) GC skew (G + C/G – C) in a 1,000-bp window. (5) rRNA (blue), tRNA (green), miscellaneous other RNA (orange), transposable elements (pink), and pseudogenes (gray).

The genome of *T. maritimum* NCIMB 2154^T^ consists of a circular chromosome of 3,435,971 bp with a 32.01% GC content. No plasmid was identified. The chromosome is predicted to contain 2,866 protein-coding genes, 6 rDNA operons, and 57 tRNA (**Figure 1**). The minimal gene set includes well-conserved housekeeping genes for basic metabolism and macromolecular synthesis, many of which are essential (the list of these genes was taken from Gil et al., 2004). Accordingly, we formally identified 203 out of the 206 protein-coding genes proposed by these authors to represent this core minimal gene set. Strikingly, the genome is rich in repeat sequences, encompassing 10.35% of the genome. Twenty-five GIs were predicted (Supplementary Table S1), two of which (GI2 and GI9) are likely from phage origin (i.e., containing elements such as a tRNA border and motility genes, and displaying a compositional bias). Genes encoding rhs family proteins and rhs (rearrangement hot-spot) associated vgr proteins (or their remnants) were frequently identified in the predicted islands. Genes encoding Cas1, Cas2, and Cas9 were identified in GI19. However, the unconventional gene organization of the *cas* genes and the lack of short direct repeats interspersed with spacer sequences strongly argues for a functionally defective CRISPR system.

### The Genome of *T. maritimum* NCIMB 2154^T^ Reveals Errors in Culture Collections

While this work was in progress, a WGS assembly of the *T. maritimum* type strain retrieved from the NBRC collection (NBRC 15946^T^; RefSeq assembly accession: GCF_000509405.1) was released but not published. Its comparison with the complete genome of strain NCIMB 2154^T^ presented in this study revealed unexpected sequence discrepancies as both cultures were supposed to represent the type strain. Using MLST (Habib et al., 2014), we confirmed that strains NCIMB 2154^T^, ATCC 43398^T^, and CIP 103528^T^ all share identical sequences for the seven loci [corresponding to Sequence Type 1 (ST1)] and are, as predicted, most likely of the same origin. In contrast, strain NBRC 15946^T^ and its derivative strain DSM 17995^T^, both possess the same sequences for the seven loci (corresponding to ST32) that differ from the sequences of ST1 at four loci (i.e., *atpA*, *dnaK*, *glyA*, and *gyrB*). According to the dates and order of deposition in the different culture collections, NCIMB 2154^T^, ATCC 43398^T^, and CIP 103528^T^ represent the *bona fide* type strain (Supplementary Figure S1).

### Metabolism

Genome analysis indicated the presence of a complete Embden–Meyerhof–Parnas pathway, of the tricarboxylic acid cycle and of genes encoding NADH dehydrogenase subunits, cytochrome *c*, cytochrome *c* oxidase, components of ATP synthase genes as well as enzymes needed to synthesize amino acids, nucleotides, fatty acids, heme, vitamins, and coenzymes (e.g., biotin, farnesyl diphosphate, coenzyme A, NAD, FAD, dihydrofolate, mevalonate, and thiamin). However, and in contrast with *T. soleae*, the cobalamin biosynthesis encoding genes are absent in the *T. maritimum* genome. Nitrate reduction (Wakabayashi et al., 1986) is likely performed by the periplasmic, cytochrome *c*-linked, nitrate reductase complex NapAB (*MARIT_1701-1700*).

A relevant characteristic of the lifestyle of pathogens is nutrient acquisition from their host. *T. maritimum* is able to degrade proteinaceous compounds (e.g., gelatin and casein) and to grow on casamino acids or tryptone as a sole carbon and nitrogen source (Wakabayashi et al., 1986). Accordingly, the *T. maritimum* genome encodes predicted secreted proteases (see below), peptide/amino acid transporters and peptide/amino acid catabolic pathways likely involved in protein degradation and uptake from host tissues. *T. maritimum* has been reported to be unable to degrade most simple and more complex carbohydrates (Wakabayashi et al., 1986; Avendaño-Herrera et al., 2006b). However, sugar transporters and CAZymes were predicted. Overall, the genome of *T. maritimum* encodes 59 CAZymes encompassing 18 glycosyl hydrolase, 30 glycosyl transferases, one polysaccharide lyase, six carbohydrate binding modules containing proteins, and four carbohydrate esterases (Supplementary Table S2).

Polysaccharide utilization loci (PUL), restricted to and very common within the phylum Bacteroidetes, are gene clusters involved in the capture, degradation, and import of complex carbohydrates. PUL-encoded proteins encompass a SusD-family cell-surface lipoprotein that binds the oligosaccharide, and a SusC-family TonB-dependent receptor for its transport across the bacterial outer membrane (Anderson and Salyers, 1989). Moreover, genes encoding SusC- and SusD-family proteins are usually organized in tandem in the genomes of Bacteroidetes (Terrapon et al., 2015). In full accordance with its inability to use carbohydrates, the *T. maritimum* genome presents a very low amount of *susC/susD* pairs compared to other members of the phylum Bacteroidetes (Barbeyron et al., 2016). Moreover, among the six identified *susC*/*susD* containing loci, only one harbors a typical PUL structure (*MARIT_2678 - 2679*) and is predicted to be involved in glycan harvesting from host glycoproteins (see below).

### Iron Acquisition and Utilization

Iron acquisition from host plays an important role in virulence of many pathogenic bacteria. In biological systems, high-affinity iron-binding proteins can chelate iron, and pathogens have developed efficient mechanisms to obtain iron from their hosts (Ratledge and Dover, 2000). In the *T. maritimum* genome, we identified a siderophore biosynthesis gene cluster (*MARIT_0169-0174*) highly similar to the *mbs* locus from a deep-sea metagenome (Fujita et al., 2012). As this gene cluster is predicted to be involved in the production of the macrocyclic hydroxamate class bisucaberin siderophore, we named the genes *tbs* for *Tenacibaculum* bisucaberin synthase. However, the gene organization is different from those previously reported displaying a major facilitator-family exporter-encoding gene and a duplication/fusion of the *tbsCD* gene (Supplementary Figure S2). A highly similar locus is observed in the genome of the *Tenacibaculum mesophilum* type strain (CIP 107215^T^; data not shown), likely responsible of the bisucaberin B siderophore biosynthesis as proposed by Fujita et al. (2013). Among the numerous TonB-dependent outer membrane receptors encoded in the *T. maritimum* genome, *MARIT_0185*, located in the *tbs* locus neighborhood, likely encodes the bisucaberin siderophore-iron transporter.

In the human periodontal bacterium *Porphyromonas gingivalis*, the heme-binding lipoprotein HmuY, together with the outer-membrane receptor HmuR, are predicted to be virulence factors during bacterial infection (Olczak et al., 2008; Wójtowicz et al., 2009). In the *T. maritimum* genome, two genes (*MARIT_1312-1313*), organized in tandem, encode HmuR and HmuY homologous proteins and are predicted to be involved in heme uptake.

In addition, *MARIT_0141 – 0142* encoding FeoAB likely constitute a Fe^2+^ uptake system (Lau et al., 2016) and two iron-regulated protein homologous genes (*MARIT_1664* and *MARIT_1661*), belonging to the imelysin family, might also be involved in iron acquisition, uptake or storage (Xu et al., 2011). The control of iron metabolism is likely carried out by the ferric uptake regulator Fur (*MARIT_1835*; Fillat, 2014). Hence, such a variety of iron acquisition systems strongly suggests the ability of this bacterium to survive under poor iron conditions (sea water) and/or to retrieve iron sequestered by host proteins (Avendaño-Herrera et al., 2005b).

### Motility, Adhesion, Quorum Sensing/Quenching, and Stress Response

Like most members of the family Flavobacteriaceae, *T. maritimum* moves over surfaces by gliding motility, an active process that does not involve pili or flagella. The genome of *T. maritimum* encodes all the proteins that form the gliding machinery, i.e., the 14 *gld* genes (*gldA* to *gldN*; McBride et al., 2009) and 10 *spr* genes (*sprA*, *sprB*, *sprC*, *sprD*, *sprE*, and five *sprF* paralogs; McBride and Zhu, 2013). *T. maritimum* NCIMB 2154^T^ is extremely adherent to different surfaces including agar, plastic, and glass. Genes encoding (i) the biosynthesis of exopolysaccharides (*MARIT_2522-2537*); (ii) the numerous adhesins (*n* = 17); and (iii) the proteins displaying lectin or carbohydrate-binding motifs could be involved in these strong adhesive properties, in the biofilm-forming ability and in the hemagglutination properties of the bacterium (Pazos, 1997).

*Quorum sensing* is a bacterial communication process that controls a range of functions at the population level. In Gram-negative bacteria, the most studied *quorum sensing* system comprises the production and detection of acyl homoserine lactones (AHLs), diffusible compounds that act as signaling molecules between cells (Garg et al., 2014). Though AHL production was previously reported in *T. maritimum* (Romero et al., 2010), no homologous gene for AHL biosynthesis was detected in its genome. In contrast, *quorum quenching* refers to all processes involved in the inhibition of bacterial communication (Kalia, 2013). A *N*-acyl homoserine lactonase encoding gene (GenBank: KR232938.1) belonging to the metallo-β-lactamase family has been proposed to be the quorum quencher of *T. maritimum* (Mayer et al., 2015). However, this gene is definitively absent from the *T. maritimum* genome and one must conclude that KR232938.1 does not belong to *T. maritimum* but rather to another fish pathogen, *T. discolor* (99.77 % nucleotide sequence identity).

Pathogenic bacteria have to adapt to the changing environments between their different lifestyles and to cope with various stresses including reactive oxygen species (ROS) produced by host macrophages. The genome of *T. maritimum* encodes three superoxide dismutases (SodA, SodB, and SodC). Most bacteria possess either a manganese-dependent (SodA) or an iron-dependent (SodB) superoxide dismutase in their cytoplasm, while zinc-dependent superoxide dismutases (SodC) have been detected mostly in pathogenic bacteria (Sheng et al., 2014). These enzymes convert superoxide anions to molecular oxygen and hydrogen peroxide, to be further metabolized by catalases or peroxidases. The presence of the three types of superoxide dismutases and two catalase/peroxidase (KatA and KatG) suggests that *T. maritimum* uses a sophisticated mechanism to face up oxidative stress. In addition, three loci involved in bacterial resistance to heavy metals have been identified: (i) *MARIT_0364-0366*, similar to the drug efflux system AcrA–AcrB–TolC of *E. coli* (Lee et al., 2012); (ii) *MARIT_1200* encoding a putative arsenate reductase; and (iii) *MARIT_1768-1771* encoding a heavy metal efflux pump-type ATPase. These loci are likely involved in the removal of cationic heavy metals to limit the production of ROS by the Fenton reaction.

### Transport and Secretion Systems

Transport systems are of great significance for virulence by addressing toxins to the bacterial surface. ABC-type transport systems, the Sec-dependent transport system, and the twin-arginine transport system were identified. In the phylum Bacteroidetes, the type IX secretion system (T9SS) allows the delivery of proteins to the cell surface (McBride and Zhu, 2013). All previously characterized components of the T9SS were identified in the *T. maritimum* genome. In *P. gingivalis*, the T9SS-secreted proteins comprise many virulence factors, including the extracellular and cell-surface cysteine proteinases gingipains (Sato et al., 2010, 2013). These T9SS-secreted proteins possess a conserved C-terminal domain (CTD), involved in secretion and cell-surface anchoring. These 70–100 amino acids long CTDs belong to two different TIGRFAM protein domain families, TIGR04183 and TIGR04131 (McBride and Nakane, 2015). The *T. maritimum* genome encompasses eight genes encoding TIGR04131-containing proteins (Supplementary Table S3). Most, if not all, are predicted to be adhesins, including SprB (*MARIT_1321*), which is also required for gliding motility (Nelson et al., 2008). In addition, several predicted toxins were identified among the 43 genes encoding TIGR04183-containing proteins.

### Toxins

**FIGURE 2:**
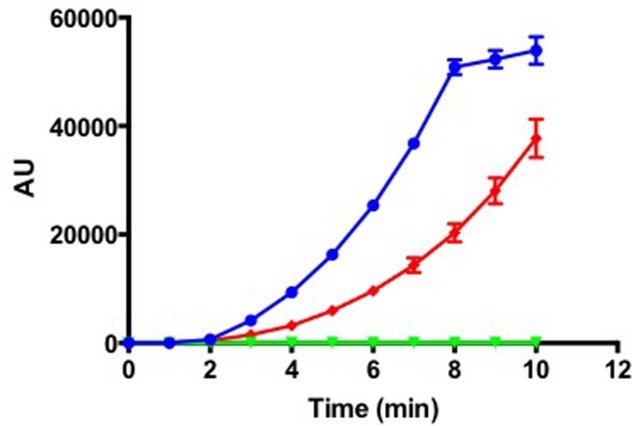
*Tenacibaculum maritimum* sphingomyelinase activity. Fluorescence measurement (arbitrary units) following incubation of 1.3 ng (blue line) of recombinant protein with Amplex Red Sphingomyelinase assay kit. The control conditions were as follows: negative control, 1.3 ng of boiled recombinant protein (green line); positive control, the purified sphingomyelinase from *Bacillus cereus* provided by the manufacturer (red line). Results correspond to the mean of triplicates and SDs are included.

As *T. maritimum* is a pathogenic bacterium, this species should possess sophisticated mechanisms to invade and colonize host tissues. Accordingly, the *T. maritimum* genome encodes a bunch of predicted toxins and virulence factors including membrane-damaging enzymes potentially involved in host cells lysis. A gene encoding a sphingomyelinase with a lipoprotein signal (*MARIT_1748*) homologous (30.2% identity/50% similarity) to the one of *B. cereus* was identified (Supplementary Figure S3A). A gene encoding a ceramidase with a signal peptide and a TIGR04183 domain (*MARIT_2033*) homologous (33.5% identity/59.8% similarity) to the one of *Pseudomonas aeruginosa* (Supplementary Figure S3B) is also present in the *T. maritimum* genome. Sphingomyelinase has been reported to be cytotoxic to host cells by acting as a potent hemolytic factor (Oda et al., 2010), while the bacterial ceramidase functions as an exotoxin or activator of exotoxin (Okino et al., 2010; Ito et al., 2014). Indeed, the outer layer of the plasma membrane of eukaryotic cells contains phospholipids, which are hydrolyzed to phosphocholine and ceramide by sphingomyelinase, the latter being subsequently hydrolyzed to sphingosine and fatty acids by a ceramidase. To formally demonstrate that *MARIT_1748* encodes the sphingomyelinase, we cloned the corresponding nucleotide sequence in the pFO4 vector (Groisillier et al., 2010). The recombinant protein was produced in a soluble form in *E. coli* BL21(*DE3*) and the enzymatic activity of the purified sphingomyelinase was assayed in triplicate using the Amplex Red Sphingomyelinase assay kit (**Figure 2**). Another predicted hemolysin is encoded by *MARIT_0124* and belongs to the cholesterol-dependent cytolysin family (Gilbert, 2010). These pore-forming toxins were originally identified in Gram-positive bacteria and encompass well-known examples including listeriolysin, perfringolysin, streptolysin, and pneumolysin (Los et al., 2013).

**FIGURE 3:**
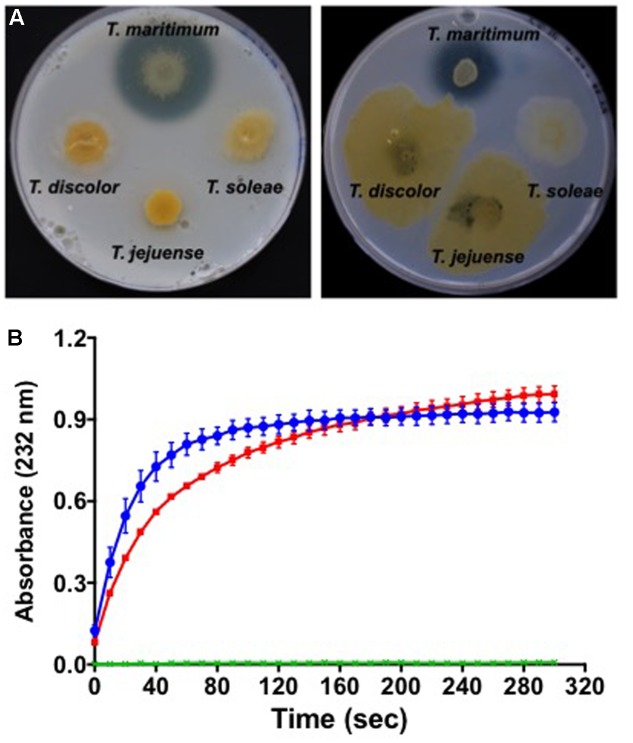
Degradation of chondroitin sulfates by *T. maritimum*. **(A)** Colonies of *T. maritimum* NCIMB 2154^T^, *T. discolor* LL04 11.1.1^T^, *T. jejuense* KCTC 22618^T^, and *T. soleae* LL04 12.1.7^T^ on marine agar 2216 supplemented with 0.2% of chondroitin sulfates A (left) and C (right). **(B)** Activity of the recombinant protein *Tm*CslA_PL8_ on chondroitin A (blue line) and chondroitin C (red line). The release of unsaturated oligosaccharides was monitored by spectrophotometry at 232 nm using biological triplicates. The two control conditions (green lines) correspond to the same reaction mixture with a boiled *Tm*CslA_PL8_. For the clarity of the graphic, the error bars are only indicated every 10 s.

Glycosaminoglycans (GAGs) are highly sulfated polymers composed of repeated disaccharide units (an amino sugar and an uronic sugar). They represent major components of animal cell surface and extracellular matrix, mostly in the form of proteoglycans. Among them, chondroitin sulfate is an important component of cartilage and fish connective tissue (Arima et al., 2013). It is composed of a chain of alternating *N*-acetylgalactosamine and glucuronic acid to which proteins attach. Chondroitin sulfate lyases have been suggested to be virulence factors, for instance in the other fish pathogen *Flavobacterium columnare* (Suomalainen et al., 2006). One might predict that the *cslA* gene (*MARIT_2107*) encoding a PL8_3 family chondroitin AC lyase highly similar to that of *F. columnare* plays a similar role. *In vitro* analyses demonstrated that *T. maritimum* is able to degrade chondroitin sulfate A and C on marine agar 2216, as showed by the formation of a degradation halo around the bacterial growth (**Figure 3A**). Although a chondroitin C-lyase activity was recently suggested for *T. maritimum* (Rahman et al., 2014), our results demonstrate the ability of this bacterium to also degrade chondroitin sulfate A. Other phylogenetically close *Tenacibaculum* species, such as *T. discolor*, *T. jejuense*, or *T. soleae* do not display this chondroitin AC lyase activity under the same conditions. To formally demonstrate that gene *cslA* encodes the chondroitin AC lyase, we cloned the nucleotide sequence corresponding to the PL8_3 catalytic module in the pFO4 vector (Groisillier et al., 2010). The recombinant protein, referred to as *Tm*CslA_PL8_, was produced in a soluble form in *E. coli* BL21(*DE3*). The enzymatic activity of the purified *Tm*CslA_PL8_ was assayed in triplicate by measuring the increase in absorbance at 232 nm of the reaction products using chondroitin A and C sulfates as substrates. As seen in **Figure 3B**, *Tm*CslA_PL8_ is highly active on both substrates, confirming the functional annotation of *MARIT_2107*. As no sulfatase could be identified in the genome, it is likely that GAGs such as chondroitin sulfate cannot be assimilated by *T. maritimum*. Therefore, CslA might be a *bona fide* virulence factor allowing the pathogen to invade fish tissues.

**FIGURE 4:**
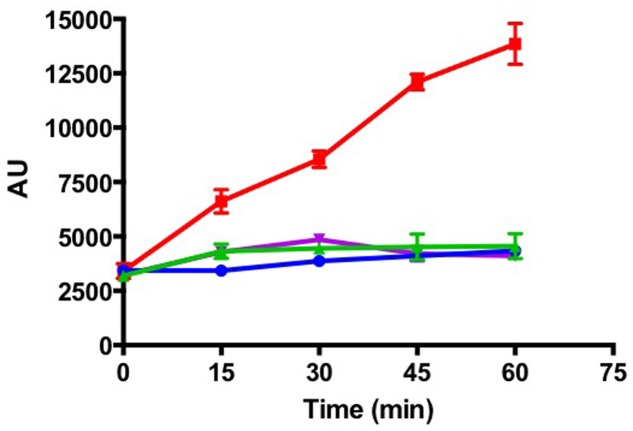
*Tenacibaculum maritimum* sialidase activity. Fluorescence measurement (arbitrary units) following incubation of *T. maritimum* NCIMB 2154^T^ (red line), *T. discolor* LL04 11.1.1^T^ (blue line), *T. jejuense* KCTC 22618^T^ (green line), and *T. soleae* LL04 12.1.7^T^ (purple line) cells with the fluorogenic substrate MUAN. Results correspond to the mean of triplicates and SDs are shown.

*Capnocytophaga canimorsus*, another member of the family Flavobacteriaceae, is a commensal of cat and dog mouth that can cause dramatic infections in bitten humans (Pers et al., 1996). *C. canimorsus* has the unusual property to feed directly on cultured mammalian cells by harvesting the glycan moiety of cellular glycoproteins, a property dependent on SiaC (Mally et al., 2008). Sialic acids are predominantly found in cell-surface exposed and secreted eukaryotic glycoproteins, being involved in many physiological, biological, and immunological functions (Varki and Varki, 2007). Mucosal surfaces are especially sialoglycan-rich and bacterial sialidases play important roles during the colonization and damage of mammalian mucosal surfaces (Lewis and Lewis, 2012). In *T. maritimum*, *siaA* (*MARIT_2686*) encodes a predicted sialidase, which activity was formally demonstrated using the fluorogenic substrate MUAN (**Figure 4**). In contrast, other *Tenacibaculum* species including the two fish pathogens *T. soleae* and *T. discolor*, for which no *siaA* homologous gene has been identified (data not shown), were unable to degrade MUAN under the same experimental conditions. Indeed, the *siaA* gene is encompassed in a [GI N° 24 inserted in a Gln-tRNA (position 2,965,888), Supplementary Table S1] and has a predicted foreign origin. The 3′ part of this GI (2,916,321–2,965,888) is mainly composed of pseudogenes including scars of transposases, Vgr family proteins and Rhs family proteins. On the other hand, the 5′ part of this GI (2,916,321–2,915,448) contains nine *bona fide* genes, predicted to encode a PUL encompassing (i) a SusC/SusD outer-membrane importer system; (ii) a *N*-acetylneuraminate lyase; (iii) a *N*-acyl-D-glucosamine 2-epimerase; and (iv) a *N*-acetylneuraminate epimerase/sodium:sialic acid symporter-fusion inner-membrane protein. This PUL system is likely dedicated to the harvesting, import, and catabolism of sialic acids from host glycoproteins (Supplementary Figure S4). Intriguingly, the sialidase displays a very unique structure including (i) a signal peptide; (ii) a carbohydrate esterase family 6 domain; (iii) a family 40 carbohydrate-binding module; (iv) two adjacent, fully duplicated, family 33 glycoside hydrolase domains; and (v) a TIGR04183 domain for T9SS-mediated secretion and cell-surface anchoring. Moreover, the predicted mechanism of glycan harvesting by *T. maritimum* is likely different from the one identified in *C. canimorsus*. Indeed, the *T. maritimum* genome is devoid of the *gpdCDGEF* operon encoded by the *C. canimorsus* PUL5 and involved in deglycosylation and import of N-linked oligosaccharides (Renzi et al., 2011). In addition, the *C. canimorsus* sialidase is a periplasmic-exposed lipoprotein that processes oligosaccharides after SucC/SusD-mediated import, whereas the *T. maritimum* sialidase is predicted to be cell-surface exposed, therefore directly processing sialic acid from glycoproteins. Hence, one might predict that both species perform the same function (foraging host glycoproteins) using common strategies (sialidase, PUL) but in sequentially different ways.

Extracellular proteases may exhibit a wide range of virulence potentials when interacting with the host defense mechanisms and tissue components. Furthermore, they may promote the survival of pathogens under adverse environmental conditions encountered in the infected host (Dubin, 2002). Since early studies, *T. maritimum* was shown to be proteolytic (Wakabayashi et al., 1986) and its proteases were suspected to act synergistically with other virulence factors, leading to tissue destruction and mortality (Baxa et al., 1988). Eight cell-surface-exposed, TIGR04183 domain-containing proteases were predicted (Supplementary Table S3), two of which likely of importance. *MARIT_2328* encodes a multi-domain protein encompassing a C10 family peptidase, highly similar to streptopain (SpeB), an important streptococcal virulence factor likely playing a role in bacterial colonization, invasion, and inhibition of wound healing (Nelson et al., 2011). *MARIT_1085* encodes a collagenase similar to that of *Cytophaga* sp. strain L43-1 (Sasagawa et al., 1995).

## Conclusions and Perspectives

We report here the complete genome sequence of *T. maritimum*, a serious pathogen of marine fish in many geographical areas. *T. maritimum* shows a lack of host specificity, affecting a variety of wild and farmed fish species (Avendaño-Herrera et al., 2006b). Sequence analysis has revealed a combination of strategies that probably confers *T. maritimum* the ability to invade, colonize, and degrade fish tissues and to exploit some cellular compounds for growth. The central metabolism of *T. maritimum* is similar to that of the other flavobacteria sequenced to date (e.g., several *Flavobacterium*, *Gramella*, *Dokdonia*, and *Polaribacter* species). However, *T. maritimum* does not possess a proteorhodopsin-encoding gene as identified in close relatives such as *Polaribacter*, *Dokdonia*, or *Psychroflexus* species, suggesting the inability of this bacterium to use light to generate proton motive force. Comparison with the available genomes of the three other fish-pathogenic *Tenacibaculum* species *Tenacibaculum dicentrarchi*, *Tenacibaculum ovolyticum*, and *T. soleae* (Grothusen et al., 2016; Lujan et al., 2016; Teramoto et al., 2016), has revealed striking differences in virulence strategies as most, if not all, the aforementioned predicted toxins (**Table 1**) are absent from the genomes of the latter species. These elements point to very different paths in the evolution of virulence as suggested using a subset of core-genome genes (Habib et al., 2014). The genome sequence of *T. maritimum* provides insights into the lifestyle of this poorly studied pathogen and may help in the development of efficient control strategies in fish farms. Indeed, the predicted virulence factors could lead to the development of attenuated *T. maritimum* variants for vaccine development. The genome of the type strain may also serve as a reference for future genomic comparisons for a better understanding of intraspecies and intragenus diversity and evolution as well as whole genome-based molecular epidemiology studies (Bayliss et al., 2017).

**Table 1:** Summary of the predicted virulence-associated genes identified in this study in the genome of *T. maritimum* NCIMB 2154^T^.

Label	Predicted function	Activity present in the following species	Activity suspected in *T. maritimum*
**Iron uptake**			
*MARIT_0169-0174*	Siderophore biosynthesis system, Tbs	Deep sea metagenome (Fujita et al., 2012)	Avendaño-Herrera et al., 2005b
*MARIT_1312-1313*	Heme uptake mechanism, HmuYR	*Porphyromonas gingivalis* (Olczak et al., 2008; Wójtowicz et al., 2009)	Avendaño-Herrera et al., 2005b
*MARIT_0141 – 0142*	Fe^2+^ uptake system, FeoAB	*Porphyromonas gingivalis* (Lau et al., 2016)	Avendaño-Herrera et al., 2005b
*MARIT_1664*	Iron acquisition, uptake or storage, imelysin family protein	*Synechococcus elongatus* (Xu et al., 2011)	Avendaño-Herrera et al., 2005b
*MARIT_1661*	Iron acquisition, uptake or storage, imelysin family protein	*Synechococcus elongatus* (Xu et al., 2011)	Avendaño-Herrera et al., 2005b
**Stress resistance**			
*MARIT_3105*	Superoxide dismutase [Mn/Fe], SodA	*Staphylococcus carnosus* (Barrière et al., 2001)	Suzuki et al., 2001
*MARIT_1670*	Superoxide dismutase [Fe], SodB	*Legionella pneumophila* (Amemura-Maekawa et al., 1996)	Suzuki et al., 2001
*MARIT_1821*	Superoxide dismutase [Cu–Zn], SodC	*Schistosoma mansoni* (da Silva et al., 1992)	Suzuki et al., 2001
*MARIT_0946*	KatG catalase	*Geobacillus stearothermophilus* (Singh et al., 2008)	Suzuki et al., 2001
*MARIT_2408*	KatA catalase	*Pseudomonas aeruginosa* (Brown et al., 1995)	Suzuki et al., 2001
**Toxins**			
*MARIT_0124*	Cholesterol-dependent cytolysin	*Capnocytophaga canimorsus* (Gilbert, 2010)	Baxa et al., 1988
*MARIT_1085*	Collagenase	*Cytophaga* sp. (Sasagawa et al., 1995)	Baxa et al., 1988
*MARIT_1748*	Sphingomyelinase	*Bacillus cereus* (Oda et al., 2010)	
*MARIT_2033*	Ceramidase	*Pseudomonas aeruginosa* (Ito et al., 2014)	
*MARIT_2107*	Chondroitin AC lyase	*Flavobacterium columnare* (Suomalainen et al., 2006)	Rahman et al., 2014
*MARIT_2328*	Streptopain family protease	*Streptococcus pyogenes* (Nelson et al., 2011)	Wakabayashi et al., 1986; Baxa et al., 1988
*MARIT_2678 - 2687*	Sialoglycan degradation and uptake	*Capnocytophaga canimorsus* (Mally et al., 2008)	


## Author Contributions

DP-P performed genome annotation, phenotypic characterization, and drafting the manuscript; AL, AR, and CL-R performed DNA extraction, library construction, and sequencing; GhM performed the optical-mapping; ZR participated in genomic data analysis; RL, TB, AG, and GuM performed gene cloning, protein expression, and biochemical characterization with substantial intellectual contribution; J-FB substantial intellectual contribution throughout the study, data analysis, and manuscript preparation. ED substantial intellectual contribution throughout the study, gene mining, interpretation of data, manuscript preparation, and responsible for acquisition of funding. All authors read and approved the final manuscript.

## Conflict of Interest Statement

The authors declare that the research was conducted in the absence of any commercial or financial relationships that could be construed as a potential conflict of interest.

## Footnotes

1 http://www.genoscope.cns.fr/agc/mage/

2 http://csbl.bmb.uga.edu/dbCAN/index.php
