# Supplementary material for: The Complete Genome Sequence of the Fish Pathogen Tenacibaculum maritimum Provides Insights into Virulence Mechanisms
Source: Front Microbiol. 2017 Aug 16;8:1542. doi: 10.3389/fmicb.2017.01542 (PMC5561996; doi:10.3389/fmicb.2017.01542)
Supplement: Supplementary file 1 [file Table_1.DOCX]

**Supplementary Table 1**: Predicted genomic islands (GI).

| Label | Begin | End | Length | Feature | nbr of predicted CDS |
| --- | --- | --- | --- | --- | --- |
| GI1 | 63156 | 97391 | 34235 | tRNA border | 25 |
| GI2 | 360530 | 382020 | 21490 | tRNA border+integrase | 16 |
| GI3 | 455772 | 471292 | 15520 |  | 17 |
| GI4 | 516395 | 531476 | 15081 |  | 16 |
| GI5 | 571179 | 587135 | 15956 | tRNA border | 14 |
| GI6 | 696813 | 727942 | 31129 |  | 35 |
| GI7 | 818344 | 840045 | 21701 |  | 19 |
| GI8 | 851752 | 879285 | 27533 |  | 23 |
| GI9 | 928330 | 957648 | 29318 | tRNA border+integrase | 31 |
| GI10 | 1072153 | 1091983 | 19830 |  | 14 |
| GI11 | 1105158 | 1112867 | 7709 | tRNA border | 5 |
| GI12 | 1139167 | 1158439 | 19272 |  | 20 |
| GI13 | 1189396 | 1228741 | 39345 |  | 42 |
| GI14 | 1511151 | 1518724 | 7573 |  | 10 |
| GI15 | 1676634 | 1698617 | 21983 |  | 15 |
| GI16 | 1909860 | 1928847 | 18987 |  | 7 |
| GI17 | 1950625 | 1959341 | 8716 |  | 7 |
| GI18 | 2069502 | 2100324 | 30823 |  | 26 |
| GI19 | 2126059 | 2176889 | 50831 |  | 36 |
| GI20 | 2216273 | 2234260 | 17988 |  | 16 |
| GI21 | 2237972 | 2247943 | 9972 |  | 8 |
| GI22 | 2456093 | 2470601 | 14509 |  | 10 |
| GI23 | 2582335 | 2643511 | 61177 |  | 43 |
| GI24 | 2894754 | 2965888 | 71135 | tRNA border | 55 |
| GI25 | 3127049 | 3154670 | 27622 |  | 33 |
