# Supplementary material for: The Complete Genome Sequence of the Fish Pathogen Tenacibaculum maritimum Provides Insights into Virulence Mechanisms
Source: Front Microbiol. 2017 Aug 16;8:1542. doi: 10.3389/fmicb.2017.01542 (PMC5561996; doi:10.3389/fmicb.2017.01542)
Supplement: Supplementary file 2 [file Table_2.DOCX]

**Supplementary Table 2**: Predicted carbohydrate active enzymes (CAZymes).

| Label | Gene | Begin | End | | Product |
| --- | --- | --- | --- | --- | --- |
| **Glycoside hydrolases (GH)** | | | | | |
| *MARIT_0104* |  | 4875 | 97391 | | Putative glycoside hydrolase, family GH74 |
| *MARIT_0241* | *lysM* | 245607 | 247094 | | Lytic murein transglycosylase, family GH23-CBM50 |
| *MARIT_0368* |  | 390715 | 394131 | | Glycoside hydrolase, family GHnc |
| *MARIT_0520* |  | 543756 | 544538 | | Glycoside hydrolase, family GHnc |
| *MARIT_0763* |  | 773863 | 774654 | | Endo-beta-N-acetylglucosaminidase-CBM50-containing protein, family GH73-CBM50 |
| *MARIT_1118* |  | 1164228 | 1165043 | | Endo-alpha-1,4-polygalactosaminidase, family GH114 |
| *MARIT_1122* |  | 1167983 | 1170904 | | Beta-*N*-acetylhexosaminidase / beta-lactamase-like protein, family GH3 |
| *MARIT_1314* |  | 1373332 | 1374636 | | Glycoside hydrolase, family GH99 |
| *MARIT_1473* |  | 1559242 | 1560927 | | Chitinase D, family GH18 (fragment) |
| *MARIT_1474* |  | 1560936 | 1562561 | | Chitinase D, family GH18 (fragment) |
| *MARIT_1475* |  | 1562558 | 1563481 | | Chitinase D, family GH18 (fragment) |
| *MARIT_1799* |  | 1936782 | 1938296 | | Glycoside hydrolase, family GH5_42 |
| *MARIT_1936* |  | 2079078 | 2080367 | | Peptidoglycan lytic transglycosylase, family GH104 |
| *MARIT_1950* |  | 2088931 | 2090295 | | Peptidoglycan lytic transglycosylase, family GH104 |
| *MARIT_1952* |  | 2091185 | 2092597 | | Peptidoglycan lytic transglycosylase, family GH104 |
| *MARIT_2218* |  | 2387132 | 2388037 | | Lytic murein transglycosylase, family GH23 |
| *MARIT_2237* |  | 2402862 | 2404625 | | Peptidase M28 / glycoside hydrolase, family GH114 |
| *MARIT_2686* | *siaA* | 2905779 | 2912534 | | Multimodular sialidase/Sialate O-acetylesterase/sialidase, family GH33 |
| *MARIT_2945* |  | 3160063 | 3161070 | | Beta-1,4-mannanase, family GH113 |
| **Glycosyltransferases (GT)** | | | | | |
| *MARIT_0245* |  | 249620 | 251920 | | Penicillin-binding protein 1A, family GT51 |
| *MARIT_0277* |  | 290124 | 290936 | | Glycogen synthase, family GT5 |
| *MARIT_0308* |  | 331399 | 332646 | | 3-deoxy-D-manno-octulosonic-acid transferase, family GT30 |
| *MARIT_0522* |  | 546131 | 547639 | | Glycosyltransferase, family GT4 |
| *MARIT_0537* | *murG* | 564165 | 565259 | | UDP-*N*-acetylglucosamine-*N*-acetylmuramyl-(pentapeptide) pyrophosphoryl-undecaprenol *N*-acetylglucosamine transferase, family GT28 |
| *MARIT_0784* |  | 797682 | 798914 | | Glycosyltransferase, family GT4 |
| *MARIT_0886* |  | 900297 | 901175 | | Glycosyltransferase, family GT2 |
| *MARIT_0888* |  | 901539 | 902624 | | Glycosyltransferase, family GT4 |
| *MARIT_1134* |  | 1185033 | 1186223 | | Glycosyltransferase, family GT4 |
| *MARIT_1135* |  | 1186220 | 1187488 | | Glycosyltransferase, family GT4 |
| *MARIT_1479* |  | 1568188 | 1569144 | | Glycosyltransferase, family GT2 |
| *MARIT_1539* | *ypjH* | 1631382 | 1632509 | | *N*-acetyl-alpha-D-glucosaminyl L-malate synthase, family GT4 |
| *MARIT_1552* |  | 1648434 | 1650212 | | Bimodular glycosyltransferase, family GT2 |
| *MARIT_1559* |  | 655513 | 1656271 | | Non-classified, family GTnc |
| *MARIT_1679* |  | 1785320 | 1786363 | | Glycosyltransferase, family GT2 |
| *MARIT_1780* |  | 1899898 | 1902288 | | Penicillin-binding protein 1C, family GT51 |
| *MARIT_1796* |  | 1930847 | 1932055 | | response regulator receiver / glycosyltransferase, family GT2 |
| *MARIT_1802* |  | 1939686 | 1940894 | | Glycosyltransferase, family GT4 |
| *MARIT_1862* |  | 1998280 | 1999230 | | Glycosyltransferase, family GT2 |
| *MARIT_2248* |  | 2415963 | 2416637 | | Glycosyl transferase, family GT2 (N-ter fragment) |
| *MARIT_2389* |  | 2550527 | 2551651 | | Glycosyltransferase, family GT4 |
| *MARIT_2577* | *lpxB* | 2787894 | 2788997 | | Lipid-A-disaccharide synthase, family GT19 |
| *MARIT_2950* |  | 3165538 | 3166230 | | Glycosyltransferase, family GT2 |
| *MARIT_2951* |  | 3166231 | 3167721 | | Glycosyltransferase, family GT2 |
| *MARIT_2984* |  | 3206758 | 3207750 | | Lipopolysaccharide core biosynthesis mannosyltransferase LpcC, family GT4 |
| *MARIT_3019* |  | 3249697 | 3250419 | | Dolichol phosphate mannosyl transferase, family GT2 |
| *MARIT_3154* |  | 3400357 | 3401154 | | Glycosyltransferase, family GT2 |
| *MARIT_3157* |  | 3403425 | 3404489 | | Glycosyltransferase, family GT4 |
| *MARIT_3159* |  | 3405815 | 3406933 | | Glycosyltransferase, family GT4 |
| *MARIT_3160* |  | 3406930 | 3408285 | | Glycosyltransferase |
| *MARIT_3161* |  | 3408286 | 3409047 | | Glycosyltransferase, family GT2 |
| **Polysaccharide lyases (PL)** | | | | | |
| *MARIT_2107* | *cslA* | 2258280 | 2260721 | | Chondroitinase-AC precursor containing a C-terminal secretion signal, family PL8_3 |
| **Carbohydrate-binding modules (CBM)** | | | | | |
| *MARIT_0153* |  | 148063 | 150147 | | CBM32-containg protein |
| *MARIT_0890* |  | 904074 | 905699 | | CBM50-containing protein |
| *MARIT_1472* |  | 1558559 | 1559107 | | CBM50-containing protein |
| *MARIT_1474* |  |  |  | | Chitin-binding protein, family CBM5 |
| *MARIT_2293* |  | 2451516 | 2453987 | | DOMON-like protein, family CBMnc |
| *MARIT_3044* |  | 3278948 | 3279463 | | BON-LysM-containing protein, family CBM50 |
| **Carbohydrate esterases (CE)** | | | | | |
| *MARIT_1502* | *lpxC/fabZ* | 1590266 | | 1591657 | Bifunctional enzyme LpxC/FabZ [Includes: UDP-3-O-[3-hydroxymyristoyl] *N*-acetylglucosamine deacetylase, family CE11 ; 3-hydroxyacyl-[acyl-carrier-protein] dehydratase FabZ], family CE11 |
| *MARIT_1908* |  | 2049399 | | 2051891 | Carbohydrate esterase, family CE14 |
| *MARIT_2146* |  | 2305544 | | 2306260 | Carbohydrate esterase, family CE14 |
| *MARIT_2683* |  | 2904118 | | 2904810 | Partial carbohydrate esterase, family CE12 |
| **Auxiliary activities (AA)** | | | | | |
| *MARIT_0946* | *katG* | 971380 | | 973581 | Peroxidase/catalase HPI, family AA2 |
