# Supplementary material for: The Complete Genome Sequence of the Fish Pathogen Tenacibaculum maritimum Provides Insights into Virulence Mechanisms
Source: Front Microbiol. 2017 Aug 16;8:1542. doi: 10.3389/fmicb.2017.01542 (PMC5561996; doi:10.3389/fmicb.2017.01542)
Supplement: Supplementary file 3 [file Table_3.DOCX]

**Supplementary Table 3**: Predicted proteins secreted by the T9SS containing the TIGR04183 or TIGR04183 domains.

| Label | Gene | Begin | End | Product |
| --- | --- | --- | --- | --- |
| TIGR04183 |  |  |  |  |
| *MARIT_0104* |  | 94875 | 97391 | Putative glycoside hydrolase, family GH74 |
| *MARIT_0153* |  | 148063 | 150147 | CBM32-containg protein, family CBM32 |
| *MARIT_0172* | *tbsCD* | 164687 | 167104 | Siderophore biosynthesis protein TbsCD |
| *MARIT_0174* | *tbsD* | 167698 | 169518 | Siderophore biosynthesis protein TbsD |
| *MARIT_0368* |  | 390715 | 394131 | Glycoside hydrolase, family GHnc |
| *MARIT_0404* |  | 423128 | 426388 | Protein of unknown function precursor containing a C-terminal secretion signal. Putative adhesin |
| *MARIT_0558* |  | 583270 | 586743 | Protein of unknown function precursor containing a C-terminal secretion signal |
| *MARIT_0780* |  | 792380 | 794188 | Protein of unknown function precursor containing a C-terminal secretion signal |
| *MARIT_0781* |  | 794392 | 795525 | Protein of unknown function precursor containing a C-terminal secretion signal |
| *MARIT_0895* | *porU* | 910439 | 913828 | Por secretion system protein PorU precursor |
| *MARIT_1019* |  | 1064675 | 1069807 | Protein of unknown function precursor containing a C-terminal secretion signal. Putative adhesin |
| *MARIT_1085* |  | 1131874 | 1135629 | Collagenase precursor containing a C-terminal secretion signal |
| *MARIT_1397* |  | 1474979 | 1475962 | Protein of unknown function precursor containing a C-terminal secretion signal |
| *MARIT_1420* |  | 1498482 | 1498799 | Protein of unknown function precursor containing a C-terminal secretion signal |
| *MARIT_1540* |  | 1632556 | 1634412 | Protein of unknown function precursor containing a C-terminal secretion signal. Putative endonuclease |
| *MARIT_1541* |  | 1634530 | 1637106 | Protein of unknown function precursor containing a C-terminal secretion signal. Putative endonuclease |
| *MARIT_1635* |  | 1731566 | 1733740 | Protein of unknown function precursor containing a C-terminal secretion signal. Putative endonuclease |
| *MARIT_1777* |  | 1894400 | 1896403 | Protein of unknown function precursor containing a C-terminal secretion signal |
| *MARIT_1778* |  | 1896569 | 1899313 | Protein of unknown function precursor containing LLR and a C-terminal secretion signal. Putative adhesin |
| *MARIT_1816* |  | 1950625 | 1953381 | Putative Metallo-dependent phosphatase containing a C-terminal secretion signal |
| *MARIT_1922* |  | 2067347 | 2068123 | Protein of unknown function precursor containing a C-terminal secretion signal |
| *MARIT_1925* |  | 2069502 | 2073260 | Protein of unknown function precursor containing a C-terminal secretion signal. Putative adhesin |
| *MARIT_2022* |  | 2163609 | 2168165 | Protein of unknown function precursor containing a C-terminal secretion signal. Putative adhesin |
| *MARIT_2028* |  | 2172837 | 2176889 | Putative lectin/glycoside hydrolase precursor containing a C-terminal secretion signal |
| *MARIT_2033* |  | 2179857 | 2182127 | Ceramidase precursor containing a C-terminal secretion signal |
| *MARIT_2045* |  | 2193531 | 2198315 | Probable M12B family metalloprotease precursor containing a C-terminal secretion signal |
| *MARIT_2055* |  | 2205926 | 2207548 | Probable S8 family protease precursor containing a C-terminal secretion signal |
| *MARIT_2107* | *cslA* | 2258280 | 2260721 | Chondroitinase-AC precursor containing a C-terminal secretion signal, family PL8_3 |
| *MARIT_2135* |  | 2291161 | 2295870 | Protein of unknown function precursor containing a C-terminal secretion signal |
| *MARIT_2328* |  | 2490861 | 2493029 | Probable C10 family peptidase, streptopain, precursor containing a C-terminal secretion signal |
| *MARIT_2396* |  | 2558952 | 2561825 | Protein of unknown function precursor containing a C-terminal secretion signal |
| *MARIT_2463* |  | 2638353 | 2640785 | Protein of unknown function precursor containing a C-terminal secretion signal |
| *MARIT_2464* |  | 2641076 | 2643511 | Protein of unknown function precursor containing a C-terminal secretion signal |
| *MARIT_2494* |  | 2674551 | 2683409 | Protein of unknown function precursor containing a C-terminal secretion signal. Putative adhesin |
| *MARIT_2507* |  | 2695605 | 2699594 | Probable M14 family carboxypeptidase precursor containing a C-terminal secretion signal |
| *MARIT_2518* |  | 2713263 | 2717189 | Protein of unknown function precursor containing a C-terminal secretion signal. Putative adhesin |
| *MARIT_2575* |  | 2786161 | 2787285 | Protein of unknown function precursor containing a C-terminal secretion signal |
| *MARIT_2638* |  | 2856645 | 2859617 | Probable M12B family metalloprotease precursor containing a C-terminal secretion signal |
| *MARIT_2686* | *siaA* | 2905779 | 2912534 | Multimodular sialidase/Sialate O-acetylesterase/sialidase, family GH33 |
| *MARIT_2972* |  | 3194646 | 3196973 | Protein of unknown function precursor containing a C-terminal secretion signal |
| *MARIT_3056* |  | 3288548 | 3290470 | M1 family metalloprotease precursor |
| *MARIT_3113* |  | 3346061 | 3349222 | Protein of unknown function precursor containing a C-terminal secretion signal |
| *MARIT_3129* |  | 3367280 | 3370753 | Putative peptidase family M4 (modular protein) |
| *MARIT_3130* |  | 3371008 | 3373692 | Probable M43 family metalloprotease |
| *MARIT_3183* |  | 3432298 | 3434910 | Protein of unknown function precursor containing a C-terminal secretion signal. Putative adhesin |
| TIGR04131 |  |  |  |  |
| *MARIT_0296* |  | 307873 | 317265 | Protein of unknown function precursor containing a C-terminal secretion signal. Putative adhesin |
| *MARIT_1205* |  | 1251109 | 1262787 | Protein of unknown function precursor containing a C-terminal secretion signal. Putative adhesin |
| *MARIT_1321* | *sprB* | 1381897 | 1399680 | Putative adhesin precursor SprB |
| *MARIT_1464* |  | 1544864 | 1551499 | Protein of unknown function precursor containing a C-terminal secretion signal. Putative adhesin |
| *MARIT_1794* |  | 1922356 | 1928847 | Protein of unknown function precursor containing a C-terminal secretion signal. Putative adhesin |
| *MARIT_2290* |  | 2443351 | 2447871 | Protein of unknown function precursor containing a C-terminal secretion signal |
| *MARIT_3095* |  | 3327601 | 3328413 | Protein of unknown function precursor containing a C-terminal secretion signal |
| *MARIT_3096* |  | 3328670 | 3329977 | Protein of unknown function precursor containing a C-terminal secretion signal. Putative adhesin |
