# Supplementary material for: The Complete Genome Sequence of the Fish Pathogen Tenacibaculum maritimum Provides Insights into Virulence Mechanisms
Source: Front Microbiol. 2017 Aug 16;8:1542. doi: 10.3389/fmicb.2017.01542 (PMC5561996; doi:10.3389/fmicb.2017.01542)
Supplement: Supplementary file 4 [file Image_1.PDF]

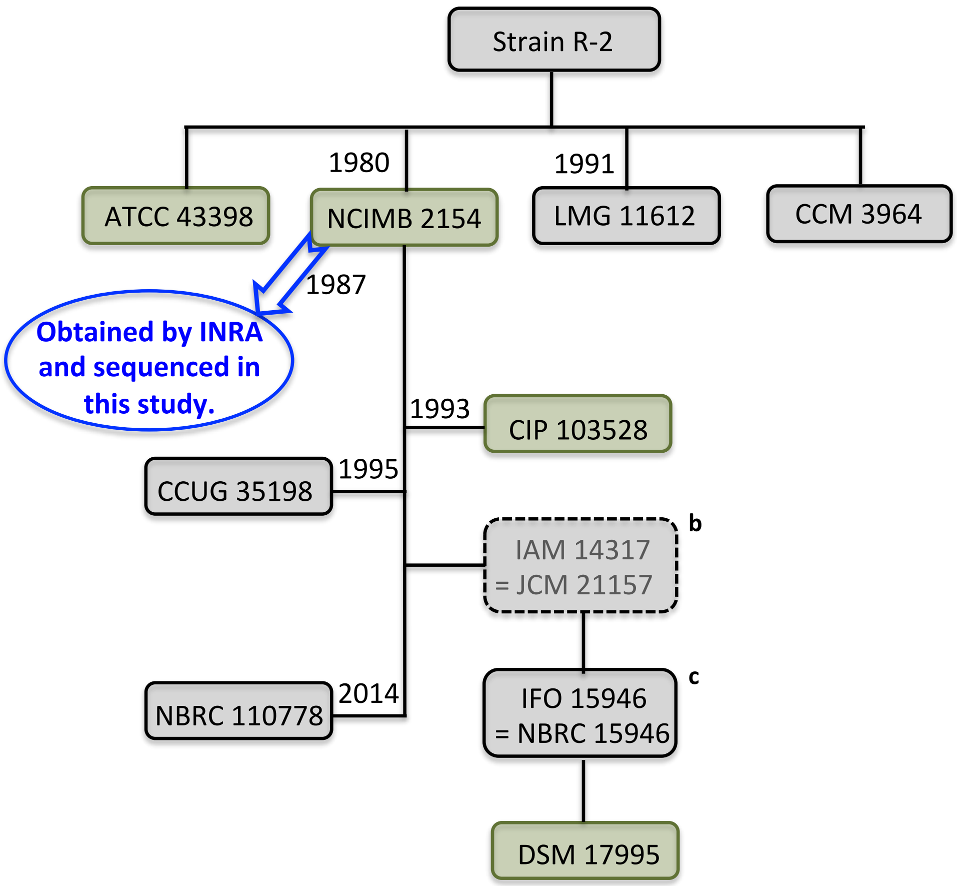

Figure S1 – Sequence of deposition of the *Tenacibaculum maritimum* type strain in different culture collections. Dates of deposition are specified when available. The different batches of the type strain that are shown in green boxes were obtained and submitted to MLST (Habib et al., 2014) in this study; the whole genome sequence of batch NBRC 15946 was retrieved from the NCBI Reference Sequence Database (RefSeq assembly accession: GCF_000509405.1). The batch of the *T. maritimum* type strain which genome was entirely sequenced in this study was obtained from NCIMB in 1987.

[a] Strain R2 was isolated in July 1977 from diseased red sea bream (*Pagrus major*) reared in Hiroshima prefecture, Japan (Wakabayashi et al., 1986).

[b] Strain numbers were changed in 2007 when the culture collection of the Institute of Molecular and Cellular Biosciences, the University of Tokyo (IAM) was transferred to the Japan Collection of Microorganisms (RIKEN BRC-JCM). These two references are no longer available (dotted box).

[c] Strain numbers were maintained in 2002 when the culture collection of the Institute for Fermentation, Osaka (IFO) was transferred to the Biological Research Center, National Institute of Technology and Evaluation (NBRC).
