# Supplementary material for: The Complete Genome Sequence of the Fish Pathogen Tenacibaculum maritimum Provides Insights into Virulence Mechanisms
Source: Front Microbiol. 2017 Aug 16;8:1542. doi: 10.3389/fmicb.2017.01542 (PMC5561996; doi:10.3389/fmicb.2017.01542)
Supplement: Supplementary file 5 [file Image_2.PDF]

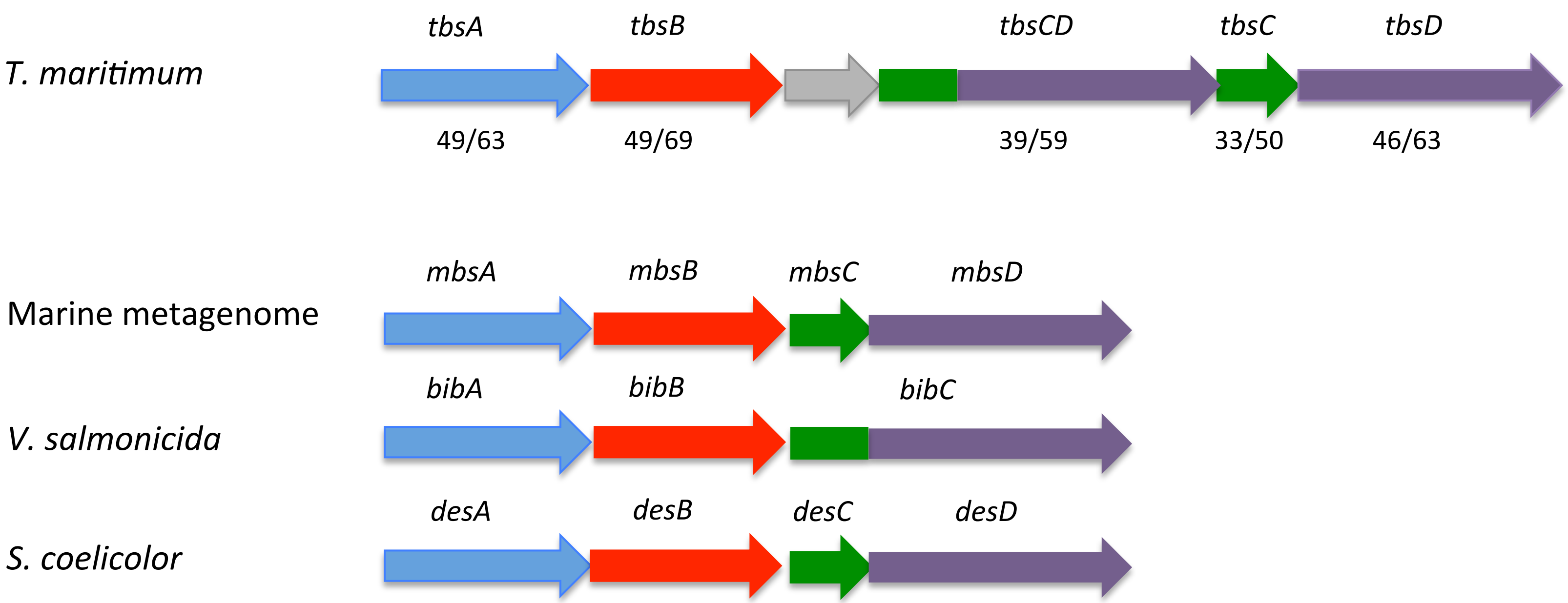

Figure S2 – ORF arrangement of the macrocyclic hydroxamate class siderophore biosynthetic gene cluters. *tbs*: gene cluster from *T. maritimum*; *mbs*: bisucaberin biosynthetic gene cluster from the marine metagenome; *bib*: bisucaberin biosynthetic gene cluster from *Vibrio salmonicida* LFI1238; *des*: desferrioxamine E biosynthetic gene cluster from *Streptococcus coelicolor* M145. Adapted from Fujita, 2012. Percentage sequence identity / similarity between Tbs and Mbs proteins are shown. The predicted major facilitator superfamily permease *TMARI1_v2_0171* is shown in grey.

- Fujita MJ, Kimura N, Yokose H, Otsuka M. 2012. *Heterologous production of bisucaberin using a biosynthetic gene cluster cloned from a deep sea metagenome*. Mol Biosyst 8:482-485.
