# Supplementary material for: The Complete Genome Sequence of the Fish Pathogen Tenacibaculum maritimum Provides Insights into Virulence Mechanisms
Source: Front Microbiol. 2017 Aug 16;8:1542. doi: 10.3389/fmicb.2017.01542 (PMC5561996; doi:10.3389/fmicb.2017.01542)
Supplement: Supplementary file 6 [file Image_3.PDF]

```
T. maritimum   MKNKLPFILMLQLLIFTGCNDDVSLGETTKEFKKRASSISTNTNITLSILSYNIFHLPSIASIYHYKEKYRAEAQKNFFN
B. cereus      MEVWNVKCKLLKGVLSLGVG----LGALYSGTSAQA-EVSTNQNDTLKVMTHNVYMLSTNLYPNW-G----QTERADLIG
S. aureus      M-VKKTKSNSLKKVATLALANLLLVGALTD-NSSKA-ESKKDDTD-LKLVSHNVYMLSTVLYPNW-G----QYKRADLIG
                         *: :   .      :*   .  . :* . ..: .  *.::::*:: *.:               : :::.

T. maritimum   TREISENMDIIIVQEAFNRY--VPIISNGLTSFINQSGLVGLYCGSWTMNRSDNYFDKMSSLSNCSNSPFVTNGGVKIYS
B. cereus      AADYIKNQDVVILNEVFDNSASDRLLGNLKKEYPNQTAVLGRSSGSEW--------DKT--LGNY-SSSTPEDGGVAIVS
S. aureus      QSSYIKNNDVVIFNEAFDNGASDKLLSNVKKEYPYQTPVLGRSQS-GW--------DKT--EGSY-SSTVAEDGGVAIVS
                 .  :* *::*.:*.*:.     ::.*  ..:  *: ::*   .           **    ..  .*.   :*** * *

T. maritimum   KWPIEYDEQLIFKNSLRGTADYLSNKGASYVRINKNGKKFHIIGTHLQADEAKKDGSGIRKLQLDELQYWIAQKIKSGKI
B. cereus      KWPIAEKIQYVFAKGCG--PDNLSNKGFVYTKIKKNDRFVHVIGTHLQAEDSMCGKTSPASVRTDQLKE-IQDFIKNKNI
S. aureus      KYPIKEKIQHVFKSGCG--FDNDSNKGFVYTKIEKNGKNVHVIGTHTQSEDSRCGAGHDRKIRAEQMKE-ISDFVKKKNI
               *:**  . * :* ..     *  ****  *.:*:**.: .*:**** *::::  .     .:: ::::  * : :*. :*

T. maritimum   PSNEPIIFAGDFNIPH-----YDIEKIKEMTTILQSNEVKLQGDLHSYDESQNTILQSNGNKYPPQTLDYILVSKQGKQP
B. cereus      PNNEYVLIGGDMNVNKINAENKNDSEYASMFKTLNASVPSYTGHTATWDATTNSIAKYNFPDSPAEYLDYIIASKDHANP
S. aureus      PKDETVYIGGDINVN------KGTPEFKDMLKNLNVNDVLYAGHNSTWDPQSNSIAKYNYPNGKPEHLDYIFTDKDHKQP
               *.:* : :.**:*:        .  :  .* . *: .     *.  ::*   *:* : *  .  .: ****:..*:  :*

T. maritimum   LFIPTFSHTTFR-------AKNTNATEDLSDHHPIKQTFYFSY
B. cereus      SYIENKVLQPKSPQWTVTSWFQKYTYNDYSDHYPVEATISM-K
S. aureus      KQLVNEVVTEKPKPWDVYAFPYYYVYNDFSDHYPIKAYS---K
                 : .                   . :* ***:*::
```

Figure S3A - Comparison of amino acid sequences of sphingomyelinase from *T. maritimum* NCIMB 2154[T], *B. cereus* ATCC 14579 and *S. aureus* RN4220. The sequences were aligned with the program ClustalW. Signal peptides are squared in red; the residues corresponding to the central metal-binding sites were retrieved from Ago *et al*., 2006 and are boxed in blues.

```
T. maritimum   M-KFQKTPRFLFILFLFIFQINYSQSNNYTIGVGISDITGQIAESAFFGYGDPFFKNGGIRDRQYARAYIVKDIHQN-SV
P. aeruginosa  MSRSAFTALLLSCVLLALSMPARADDLPYRFGLGKADITGEAAEVGMMGYSSLEQKTAGIHMRQWARAFVIEEAASGRRL
               *  :    *. :*  ::* :      ::.   * :*:* :****: **  .::**..    *..**: **:***:::::  ..  :

T. maritimum   VYVSIDKGATFQSVNLAVIEKLREAYGNLYTDTNVVISATHTHVSPGGYSHYGLYNTSTGGFYKTNFTILVDGIFNAIQQ
P. aeruginosa  VYVNTDLGMIFQAVHLKVLARLKAKYPGVYDENNVMLAATHTHSGPGGFSHYAMYNLSVLGFQEKTFNAIVDGIVRSIER
               ***. * *   **:*:* *: :*:   *  .:* :.**::*****  .*****.:** *.  ** :..*.  :****..:*::

T. maritimum   AHNNLAPGRIYYNTGSLTNASINRSLVAYNLNADANDYI-SIDDQMTVLKFIQGDTEV-GMISWFAVHPTNLSNSYKYAS
P. aeruginosa  AQARLQPGRLFYGSGELRNANRNRSLLSHLKNPDIVGYEDGIDPQMSVLSFVDANGELAGAISWFPVHSTSMTNANHLIS
               *:  .* ***::*.:*.* **. ****:::   *.*   .*   .** **:**.*:::.: *: * ****.**.*.::*: :  *

T. maritimum   GDNKGHASLKFERHKGASYGKGAATFVAAFSNSNAGDMSPNLNQPLPSDLYTNATGPGNNEEESTEIIGNRQYYKALDLY
P. aeruginosa  PDNKGYASYHWEHDVS----RKSG-FVAAFAQTNAGNISPNLNLK-------PGSGPFDNEFDNTREIGLRQFAKAYEIA
                ****:** ::*:. .     : :. *****:::***::*****           .:** :** :.*. ** **: ** ::

T. maritimum   NNAHIQLIGTIKAVSRYSDYSNILVSPKFTDGYYQSTCKAALGISFRAGA-EDGRSGIGKE-GQTRSN------PTA--G
P. aeruginosa  GQAQEEVLGELDSRFRFVDFTRLPIRPEFTDGQPRQLCTAAIGTSLAAGSTEDGPGPLGLEEGNNPFLSALGGLLTGVPP
               .:*: :::* :.:  *: *::.: : *:****  :. *.**:* *: **: ***  . :* * *:.          *.

T. maritimum   FEVDRCHMEKPIDPLFYVGANNNDPKTPKILPTSILKIGQLGILAAPAEFTVMAGRRVKATVL-ENKNTEIQYTVFAGYS
P. aeruginosa  QELVQCQAEKTI--LADTGNKKPYPWTPTVLPIQMFRIGQLELLGAPAEFTVMAGVRIRRAVQAASEAAGIRHVVFNGYA
                *: :*: **.*  *  .* ::  * **.:** .:::**** :*.********* *:: :*   .: : *::.** **:

T. maritimum   DAYSGYVTTREEYASQQYEGASTQFGPWTLAAYRQEFEKLTSILADPNVTPWDTPAPIPPRKNYIGSDKTVPILFDDIPW
P. aeruginosa  NAYASYVTTREEYAAQEYEGGSTLYGPWTQAAYQQLFVDMAVALRERL--PVETSA-IAPDLSCCQMNFQTGVVADDPYI
               :**:.**********:*:***.** :**** ***:* * .:: * :    * :*.* *.*  .   :  . :: **

T. maritimum   FKSFGSIFSNTNDNYSTDEIAEVTFWGAHPNNDPKTNSTYFRVQKKIGDSW---VSKYEDRDTNTKLIWNRDGVANSKIT
P. aeruginosa  GKSFGDVLQQPRESYRIGDKVTVAFVTGHPKNDLRTEKTFLEVVNIGKDGKQTPETVATDNDWDTQYRWERVGISASKAT
                ****.::.:..:.*  .: . *:*  .**:** :*:.*::.*::.* :   *.     :  *.* :*:  *:* *:: ** *

T. maritimum   IQFKITSDVESGHYRIIHHGKWKNGWNGKLTPYTGISNTFYIHQNFNRAVPTSKNTINNILIYPNPTSGAFTIINERLLT
P. aeruginosa  ISWSIPPGTEPGHYYIRHYGNAKNFWTQKISEIGGSTRSFEVLGTTP---------------------------------
               *.:.*....*.*** * *:*: ** *. *::   * :.:* :  .

T. maritimum   GNYSITNAMGQIVKKGKLSSKSHHLINMDVLPGMYFIKTIFQNGETNTISIVKK
P. aeruginosa  ------------------------------------------------------
```

Figure S3B – Comparison of amino acid sequences of the ceramidase from *T. maritimum* NCIMB 2154[T] and *Pseudomonas aeruginosa PAO1*. The sequences were aligned with ClustalW. *P. aeruginosa* protein motifs were retrieved from the Uniprot entry (UniProtKB: Q9I596). The signal peptides of each protein are boxed in red, the conserved amino-acids involved in metal binding are boxed in blue, those involved in substrate binding are boxed in black, the active site is boxed in green and the predicted CTD1 of *T. maritimum* is boxed in orange.
