# Supplementary material for: The Complete Genome Sequence of the Fish Pathogen Tenacibaculum maritimum Provides Insights into Virulence Mechanisms
Source: Front Microbiol. 2017 Aug 16;8:1542. doi: 10.3389/fmicb.2017.01542 (PMC5561996; doi:10.3389/fmicb.2017.01542)
Supplement: Supplementary file 7 [file Image_4.PDF]

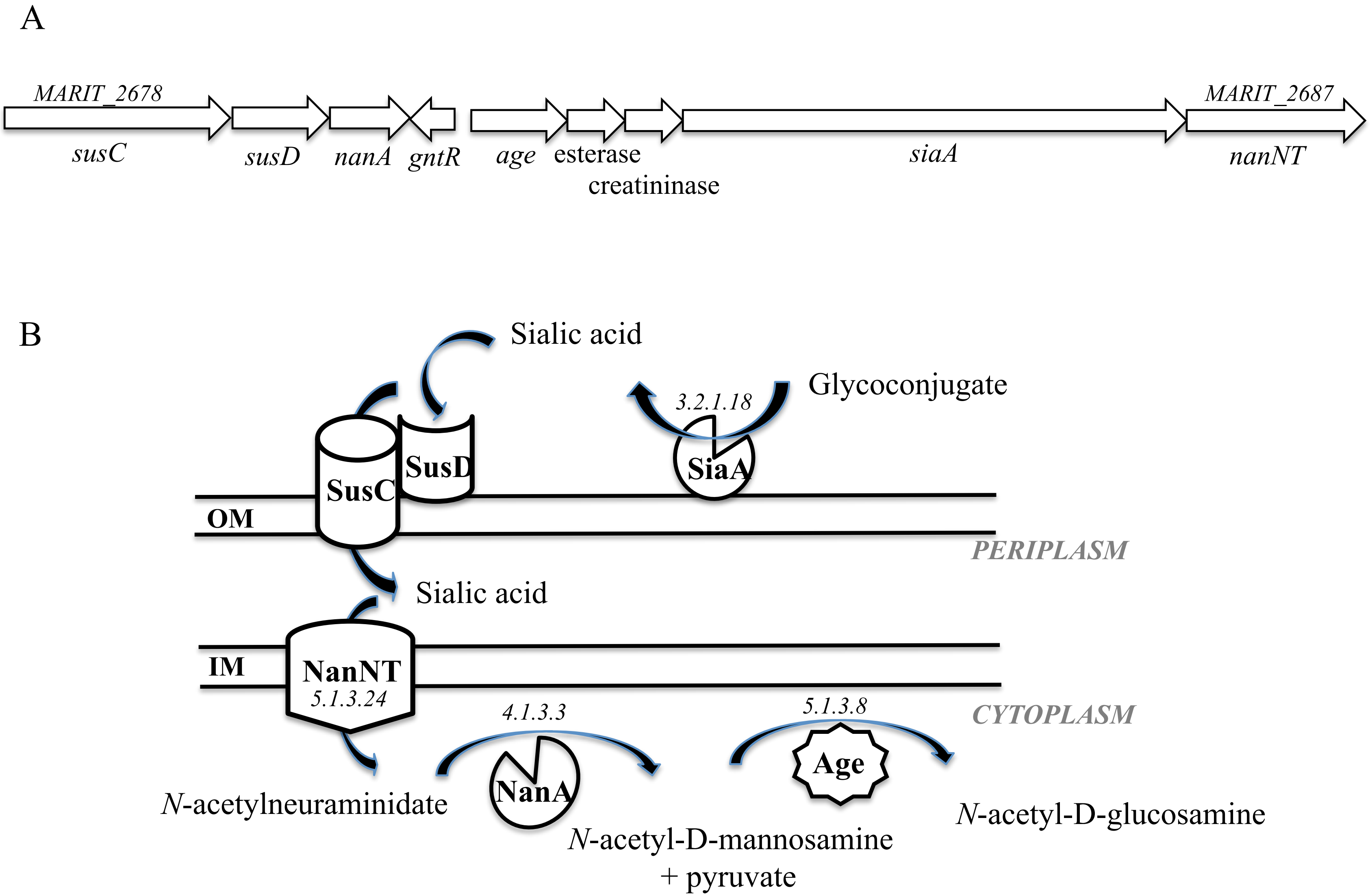

Figure S4 - (A) Schematic representation of the sialic acid utilization locus of *T. maritimum*. (B) Model of sialic acid utilization. The cell surface–anchored sialidase SiaA cleaves terminal sialic acids associated to host glycoconjugates; free sialic acid binds to the SusD-family lipoprotein to allow transport across the outer membrane by the SusC-family porin; the transport from the periplasm to the cytoplasm is driven by the bifunctional NanTN encompassing a MFS permease domain and a maturotase domain producing *N*-acetylneuraminidate; the *N*-acetylneuraminate lyase NanA cleaves the latter to produce pyruvate and *N*-acetyl-D-mannosamine which is subsequently converted to *N*-acetyl-D-glucosamine by the *N*-acyl-D-glucosamine 2-epimerase Age . E.C. numbers are indicated in italic.
